# Large-Scale Model-Based Assessment of Deer-Vehicle Collision Risk

**DOI:** 10.1371/journal.pone.0029510

**Published:** 2012-02-16

**Authors:** Torsten Hothorn, Roland Brandl, Jörg Müller

**Affiliations:** 1 Institut für Statistik, Ludwig-Maximilians-Universität München, München, Germany; 2 Fachbereich Biologie, Philipps-Universität Marburg, Marburg, Germany; 3 Sachgebiet Forschung und Dokumentation, Nationalparkverwaltung Bayerischer Wald, Grafenau, Germany; 4 Lehrstuhl für Terrestrische Ökologie, Technische Universität München, München, Germany; University of California, Berkeley, United States of America

## Abstract

Ungulates, in particular the Central European roe deer *Capreolus capreolus* and the North American white-tailed deer *Odocoileus virginianus*, are economically and ecologically important. The two species are risk factors for deer–vehicle collisions and as browsers of palatable trees have implications for forest regeneration. However, no large-scale management systems for ungulates have been implemented, mainly because of the high efforts and costs associated with attempts to estimate population sizes of free-living ungulates living in a complex landscape. Attempts to directly estimate population sizes of deer are problematic owing to poor data quality and lack of spatial representation on larger scales. We used data on 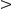
74,000 deer–vehicle collisions observed in 2006 and 2009 in Bavaria, Germany, to model the local risk of deer–vehicle collisions and to investigate the relationship between deer–vehicle collisions and both environmental conditions and browsing intensities. An innovative modelling approach for the number of deer–vehicle collisions, which allows nonlinear environment–deer relationships and assessment of spatial heterogeneity, was the basis for estimating the local risk of collisions for specific road types on the scale of Bavarian municipalities. Based on this risk model, we propose a new “deer–vehicle collision index” for deer management. We show that the risk of deer–vehicle collisions is positively correlated to browsing intensity and to harvest numbers. Overall, our results demonstrate that the number of deer–vehicle collisions can be predicted with high precision on the scale of municipalities. In the densely populated and intensively used landscapes of Central Europe and North America, a model-based risk assessment for deer–vehicle collisions provides a cost-efficient instrument for deer management on the landscape scale. The measures derived from our model provide valuable information for planning road protection and defining hunting quota. Open-source software implementing the model can be used to transfer our modelling approach to wildlife–vehicle collisions elsewhere.

## Introduction

During the last century, ungulates have gained substantial public relevance in several temperate ecosystems of Europe and North America. Especially the roe deer *Capreolus capreolus* in Europe and the white-tailed deer *Odocoileus virginianus* in North America dominate more and more the fauna of ungulates [1], [2]. Ungulate densities are relevant on larger scales because of browsing damage, which renders deer densities a major issue in farming, forestry, and conservation of biodiversity [3], [4], [5], [6]. Selective browsing by roe deer decreases the diversity of tree species during forest regeneration, which interferes with the conversion of conifer plantations to diverse forests. Browsing can even lead to the local extinction of rare tree species, such as the white fir *Abies alba* in Central Europe [4], [7], [8].

Furthermore, collisions of vehicles with either deer species are a serious threat to human health and animal welfare [for white-tailed deer, see 9]. Each year in Germany, approximately 200,000 roe deer collide with vehicles, which is almost 20% of the roe deer harvest by hunters (online at http://www.jagdschutz.de). These collisions lead to approximately 3,000 injured people, with 50 fatalities and costs of about 490 Mio. € (online at http://www.gdv.de). The number of deer–vehicle collisions (DVCs) is even expected to increase in many countries with the increase in traffic [10], [11], [9]. Most attempts at decreasing the number of DVCs through various road construction techniques (e.g., repellents, wildlife crossings, reflectors) have had little success, except for fencing [10].

Management programs that aim at decreasing DVCs and browsing damage require information on the spatial distribution of deer. However, all methods currently available for a direct estimation of deer densities, e.g., using visual methods such as thermal imaging or spotlight surveys [12], [13] or indirect measurements such as fecal counts or measuring jaw length [14], are only applicable to small areas. On larger scales, as required for state-wide management actions, these methods are rendered difficult or even impossible due to the high costs associated with these survey methods. However, Morellet et al. [2] argue that for management purposes it is not necessary to obtain absolute measures of population size. Instead, cost-effective relative indices of deer density are of considerable value for data-driven management decisions. One relative index for deer abundance is the intensity of browsing of palatable tree species. Several authors noted a close relationship of deer density and browsing intensity [15], [16], [6]. This method is attractive because one directly measures the risk of browsing on saplings, i.e., the event forest managers are primarily interested in. However, the costs associated with large-scale browsing surveys are also rather high, and the relationship between deer densities and browsing is nonlinear [16].

Given the high costs and low precision of many survey methods for estimating absolute or relative deer abundances, it is not surprising that the idea of monitoring deer densities indirectly by means of the number of DVCs was published as early as 1959 [17]. Even though the number of these accidents was quite low at that time, a positive correlation between deer density and the number of DVCs was found. More recent studies also found a positive correlation between the number of DVCs and absolute density estimates of roe deer and white-tailed deer [18], [11], [19]. The relationship between deer density and DVCs cannot, however, be assumed to be linear. In addition, the strong effects of the environment and traffic, which confound the situation, have to be taken into account [10]. DVCs have received considerable interest also in the emerging field of road ecology, e.g., for measuring the impact of traffic on species [20]. Our research does not target the interaction between roads and deer per se; instead, we consider DVCs as a simple sampling method for indirectly obtaining information on deer densities across a complex landscape.

The number of DVCs is an attractive and cost-efficient relative index for deer management. For example, in Germany, most accidents are registered because a police report is required for coverage of the car damage by insurance companies. Moreover, we can expect the bias associated with these numbers to be rather low since, unlike hunters, car drivers are not selective. DVC data is occasionally used in some regions of France [2], and models predicting the local risk of DVCs are available for white-tailed deer in Arkansas [19] and moose *Alces alces* in Newfoundland [21] and Sweden [22], but surprisingly, no state-wide monitoring system for DVCs has been implemented so far.

The first steps towards management programs that focus on a reduction in DVCs is a clear understanding of the factors that drive DVCs–separating those effects that influence the deer density and thereby the roadkills and those that affect the roadkills because of constructional reasons, such as the type of the road–and an assessment of the spatial variation of DVCs. The primary aims of our investigation are to identify the main drivers influencing the local risk of DVCs by means of a statistical model and to derive a relative index for DVC risk assessment. Since the local risk of a DVC depends on the deer density, all environmental variables that have been used to describe ungulate densities on larger scales are potentially useful as predictor variables in such a model. In general, climate and land use variables have been found to be predictive for white-tailed deer and roe deer densities. Latham et al. [23], Mysterud et al. [24], and Melis et al. [25] report a positive influence of increasing temperature on the density of these species. In contrast, precipitation, and in particular high precipitation in winter, is often negatively associated with deer densities [23], [24], [26]. Productivity and deer densities have been found to be only partially positively associated [27]. With respect to variables describing land use, the proportion of conifer forests has a positive impact on white-tailed deer density [26]. Urban and larger forested areas are associated with a higher number of DVCs [28], [11] compared to wetlands and large agriculture areas. Sufficient lengths of forest edges are an important factor for good deer habitats [19], [29]. Although a positive relationship between browsing damage and deer densities is well established [3], [5], [1], [6], we are not aware of a study relating the risk of DVCs to the browsing intensity.

Here we propose a large-scale, high-resolution model for roe-deer–vehicle collisions in Bavaria, Germany, and apply a new risk-modelling approach to roe deer management and road safety issues. Our results are based on a total of 74,650 roe-deer–vehicle collisions from Bavaria, Germany, reported on the 132,106 km of roads in the 2,270 Bavarian municipalities in 2006 and 2009. We combine variables describing climate, land use, and browsing intensity to estimate the number of DVCs that can be expected per kilometer of a specific road type. Since strong nonlinear effects of climate and land use variables on DVCs have to be expected [30], [11], [16], we allow for such complex regression functions. Furthermore, we explicitly model the spatial heterogeneity that may be present in such observational data and assess the temporal heterogeneity. We demonstrate that the model provides a sound basis to (i) accurately estimate the risk of a DVC for a specific municipality (also as a surrogate of deer density), (ii) analyze the correlation of browsing damage and the local accident risk, and (iii) identify important variables that define our “DVC index” as useful for wildlife management. In addition, we (iv) test how the DVC risk corresponds with deer harvest numbers as another relative index for deer densities in the study area.

## Materials and Methods

### Study site, data sources, and data preprocessing

Bavaria is located in southeastern Germany (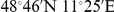, see Information S1). With an area of 70,500 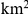, it covers almost 20% of the land area of Germany; 36% of this area, i.e., 25,000 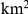, is covered by forests. Roe deer are abundant throughout Bavaria, with an annual harvest of approximately 280,000 animals [31]. In contrast, the red deer *Cervus elaphus* occurs only in special management districts, with an annual harvest of around 10,700 animals [31], and a very small population of fallow deer *Dama dama* lives in Bavaria, with an annual harvest of 400 animals.

Bavaria consists of 2,270 disjointed administrative municipalities that form the observational units of our analysis (see Information S1). The area of 47 of these municipalities is not crossed by any road; therefore, only the remaining 2,223 municipalities with roads were taken into account. Based on the official map showing the borders of the municipalities, we attributed data on roads, DVCs, climate, and land use, and information on ungulate browsing to each of these 2,223 municipalities (for technical details, see Information S1).

#### Deer–vehicle collisions

The total length of public roads in Bavaria is 132,106 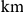 (motorways: 3,377 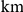; primary roads: 7,117 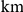; secondary roads: 14,216 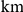; tertiary roads: 18,838 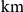; and residential streets: 88,557 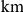). Based on the official road map, the total length of each of these five road categories was computed for each municipality. The DVC data for Bavaria from 2006 (34,129 accidents) and 2009 (40,521 accidents) were obtained from the Bavarian State Home Office (file access number IC4-3607.12-28). Accidents involving more than one vehicle were counted only once, and accidents that could not be attributed to a municipality (altogether 313 collisions) were removed. In principle, we were only interested in roe deer accidents, but the database contains all reported accidents with roe deer, red deer, and fallow deer without differentiation of the three species. However, according to figures published by the German Hunters Association (online at http://www.jagd-online.de), only 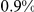 of DVCs in Bavaria involve red deer and less than 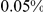 involve fallow deer. We adjusted for red deer presence in the regression analysis by including a parameter that indicates whether or not a municipality is part of a red deer management district. In general, red deer management districts consist of larger closed forest areas with a low density of roe deer [23].

The Bavarian Road Information System (online at http://www.baysis.bayern.de) was used to determine coordinates for each accident on nonresidential roads, and this location was used to determine the corresponding municipality. For residential streets, we did not determine the exact location but considered the municipality in which the accident was reported.

#### Climate and land use data

Bioclimatic variables were retrieved from the WorldClim database (online at http://www.worldclim.org) at a resolution of 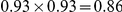 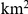 [32]. For each of the grids within a municipality, we calculated the mean of all WorldClim raster points. Based on known effects of climate on roe deer (see Introduction), we computed the following variables for each municipality: annual mean temperature (WorldClim nomenclature: bio1), temperature seasonality (bio4), minimum temperature of coldest month (bio6), temperature annual range (bio7), mean temperature of warmest quarter (bio10), mean temperature of coldest quarter (bio11), annual precipitation (bio12), precipitation of warmest quarter (bio18), and precipitation of coldest quarter (bio19).

To characterize the land use, we used the Corine 2000 map (online at http://www.corine.dfd.dlr.de) of Germany with a resolution of 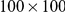 m. We combined the classifications from Corine with the following categories for each municipality: meadows, swamps, industry, urban areas, complex habitats, conifer forests, mixed forests, broadleaf forests, and arable areas (see Information S1 for detailed Corine classifications). Based on the merged forests within a municipality, we also calculated the total length of forest edges.

#### Browsing data

Bavaria is subdivided into 762 game management districts (in German “Hegegemeinschaften”, see Information S1). The amount of ungulate browsing within these districts is monitored by the Bavarian Forest Administration [33] in all of the 25,000 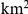 of forests in Bavaria. The survey is based on a fixed regular grid (grid length 1,250 m) that defines sampling points at its intersections. In both 2006 and 2009, at each intersection, the nearest appropriate sampling area to estimate the proportions of browsed tree saplings was selected. In each selected area, 75 saplings of 20–130 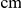 in height along a transect were examined for signs of browsing on leading shoots [34], [33], [35]. The proportion of saplings showing leading-shoot browsing is a measure commonly used to assess browsing intensity [6, among many others]. We classified saplings according to genera into four classes with respect to their palatability for roe deer [36], [7]: spruce *Picea* and pine *Pinus*; oak *Quercus* and fir *Abies*; ash/maple/elm/linden *Fraxinus/Acer/Ulmus/Tilia*; and beech *Fagus* and other hardwoods (rowan berry *Sorbus aucuparia*, birch *Betula*, alder *Alnus*, hornbeam *Carpinus betulus*, willow *Salix*, poplar *Populus*, and service tree *Sorbus domestica*). In total, our analysis of browsing is based on 3,087,231 saplings surveyed in 2006 and 2009 (see Information S1). Since not all tree genera were present in all game management districts and the number of trees in some of the four classes was rather small in certain game management districts, we used a random effects model with a Markov random field smoother to compute spatially smooth estimates of the proportion of saplings with signs of browsing (see Information S1). From this map, the browsing proportion used for each municipality was extracted as the value at the corresponding centroid of the polygon defining the municipality. The browsing survey data were made available to the first author through projects ST217 and ST230 of the Bavarian State Ministry of Food, Agriculture, and Forestry.

#### Roe deer harvests

The reported harvest for each of the game management districts for the periods 2004–2006 and 2007–2009 were obtained from the Bavarian State Ministry of Food, Agriculture, and Forestry (file access number R4-7909-1/10). In Germany, plans for roe deer management are implemented for periods of three years, and information on single years is not available.

### Statistical analysis

The number of DVCs per kilometer of a road type within a municipality in 2006 or 2009 was conditionally modelled on the environmental variables by an additive model of the form
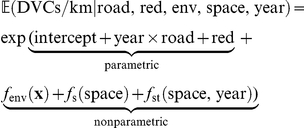
consisting of two parts. The overall intercept, the main and interaction effects of road type, and the two survey years along with the indicator (red) specifying whether the corresponding municipality is part of a red deer management district (and thus whether accidents with red deer were possible) allow us to assess the expected number of DVCs per kilometer in 2006 and 2009 for the different road types with or without red deer presence. These effects shall not be subject to variable selection or penalization in the modelling process, and we therefore treated these parametric effects as mandatory.

In contrast, the contributions of the environment and the model terms capturing spatio-temporal autocorrelation allow for smooth and nonlinear regression functions. Furthermore, we expected only a small number of these model terms to be actually relevant. Thus, variable selection and penalization of the estimated effects were crucial. Although it would have been technically possible to fit both parts of the model simultaneously, Hofner et al. [37] showed that a simultaneous model fit favors more flexible model terms and can lead to a serious variable selection bias. This phenomenon occurs when model components of different complexity are compared with respect to some goodness-of-fit criterion for variable selection. Therefore, Boulesteix and Hothorn [38] suggested to fit a model based on the parametric part of the model first, and, in a second step, to model the deviations from this initial model.

We adopted this two-step procedure and first modelled the number of DVCs by the intercept, the main and interaction effects of year and road type, and red deer presence. These 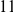 parameters were estimated based on 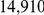 observations (525 municipalities crossed by motorways, 1,048 by primary roads, 1,724 by secondary roads, 1,977 by tertiary roads, and 2,181 by residential streets in both years) without any form of variable selection or penalization by means of a linear Poisson model with a natural link function:
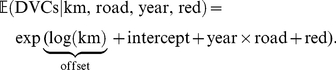
(1)The logarithm of the length of the corresponding road type within a municipality was treated as offset, i.e., the corresponding regression coefficient was fixed at one. Dividing both sides of the equation by the road length conveniently leads to a model for the average number of DVCs per kilometer for each road type in the two years with or without red deer presence. Note that unlike previously published DVC models imposing a normal distribution on the skewed number of DVCs per kilometer, we modelled the count variable DVC using the Poisson distribution and standardized it to road length after fitting the model. Model (1) was estimated using standard Poisson linear model software for maximum-likelihood estimation [R version 2.12.2, 39].

To model the deviations from this parametric model that may be due to the environment (env) or spatio-temporal heterogeneity (space, year), we applied the species distribution modelling framework introduced by Hothorn et al. [40]. The expected number of DVCs is described by an additive Poisson model with an offset defined by the parametric model (1):
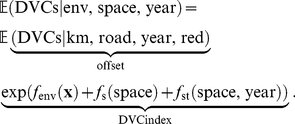
(2)We assumed that the partial contributions of the environmental variables are smooth nonparametric additive functions 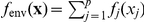, where 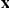 denotes the vector containing variables describing climate, land use, and browsing intensity for some municipality. Because a positive relationship between browsing intensity and deer densities is well established [e.g.], [ 3], [ 5], [ 1, 6, among others], we imposed a monotonicity restriction on the partial contributions of the browsing intensities [41]. Spatial autocorrelation present in the data was captured by a smooth bivariate function 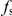 of the municipality centroids. Note that for each observation, a separate multiplicative effect 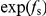 was estimated that, similar to observation-specific random intercepts in a linear mixed Poisson model, can be interpreted as an overdispersion term [42]. Spatial differences between the successive years were modelled by a smooth bivariate function 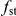 allowed to take nonzero values for observations in 2009 only [see 40]. This model component captures spatio-temporal differences and can be used to assess the effect of local management interventions. A sufficient local reduction of deer densities in one year causing a reduced number of DVCs in subsequent years will be associated with a negative estimate of 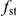.

The estimated model component 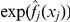 for variable 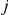 can be interpreted as the relative multiplicative change (increase or decrease) in the expected number of DVCs per kilometer caused by a value 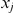 of the 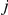
th environmental variable when all other variables remain constant. The variability of the product of groups of fitted nonparametric components (i.e., for climate, land use, and browsing variables) is a means to assess the relative importance of these groups of environmental variables in the model. The product of all nonparametric components can be interpreted as the relative multiplicative change in DVCs per kilometer for a municipality with certain environmental conditions. We introduce the term “DVC index” for the nonparametric model part as a relative index for DVC risk.

The nonparametric part was estimated by component-wise boosting as implemented in the R add-on package **mboost** [43]. In essence, boosting is a novel estimation technique for generalized additive models with a larger number of potential environmental factors, including variable selection. We refer the reader to Büehlmann and Hothorn [44] and Hothorn et al. [40] for a more detailed description. The boosting algorithm applied here fits the model by iteratively minimizing the Poisson log-likelihood by means of nonlinear functions of the environmental variables. The optimal number of boosting iterations–the main tuning parameter describing model complexity–was determined via internal validation using the bootstrap. For 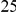 bootstrap samples of the data, the model was refitted and the out-of-bootstrap log-likelihood (i.e., a measure of model performance evaluated on independent validation data) was assessed for increasing numbers of iterations (see Information S1). Model inference was performed with the aim to select relevant variables. We applied stability selection based on subsamples of the municipalities [45], [40]. We only report results when the corresponding model component was selected with high probability (here in more than 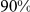 of the subsampled models) and thus when the probability that the corresponding variable is actually associated with the number of DVCs is high. Reproducible R code and data are given in the Information S1 and can be used to transfer our model to other regions and species.

In an attempt to validate our model externally, we compared the DVC index for each municipality in 2006, i.e., the term 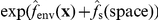, with harvest data. For each game management district, we averaged the indices of those municipalities whose centroid is in the corresponding game management district. The association between the DVC index and harvest numbers was assessed using a LOWESS smoother and Spearman's rank correlation coefficient.

## Results

**Figure 1:**
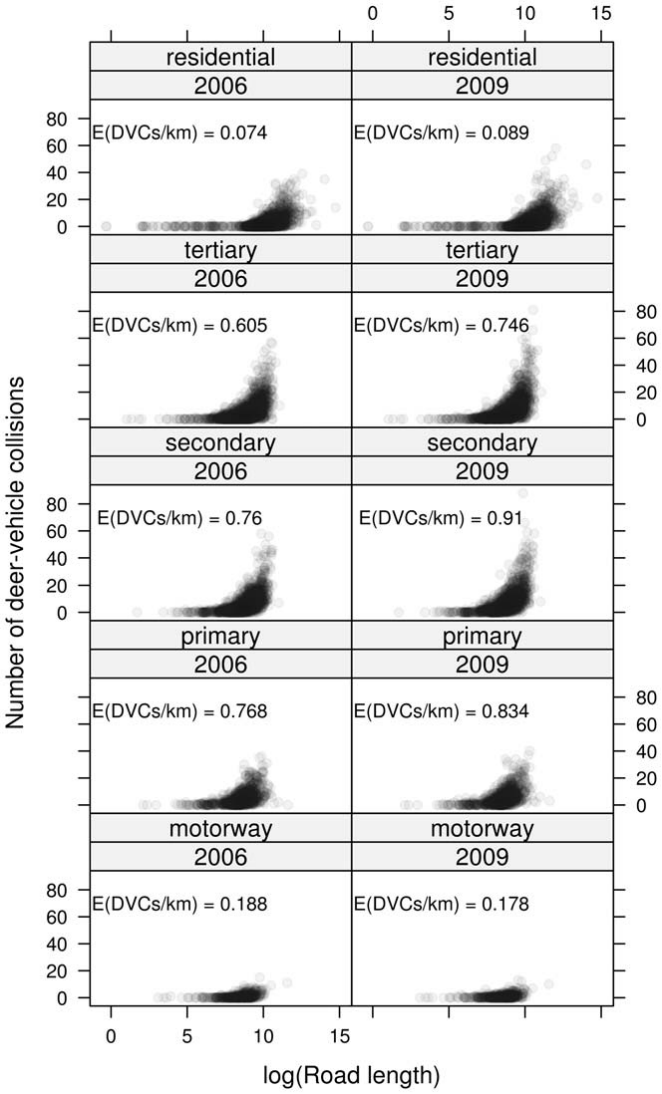
Distribution of deer–vehicle collisions. Number of DVCs for different types of roads in 2006 and 2009. Each dot represents one municipality crossed by at least one road of the corresponding type. The expected numbers of DVCs were derived from the parametric model (1).

The number of DVCs observed for one of the five road types strongly depended on the length of the road type in a municipality (Fig. 1). The shape of this relationship was similar in 2006 and 2009. Except for motorways, the number of DVCs was higher in 2009. In general, the majority of DVCs took place on secondary and tertiary roads. Therefore, it is necessary to differentiate between the different road types when modelling DVC data. The estimated number of DVCs per kilometer, obtained from the parametric model (1), ranged between 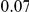 per year on residential streets in 2006 to 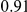 on secondary roads in 2009 (see Fig. 1). For motorways, the expected number of DVCs per kilometer was low, which is due to the fences along all motorways. For municipalities within red deer management districts, the expected number of DVCs per kilometer was reduced by a factor of 0.76.

**Figure 2:**
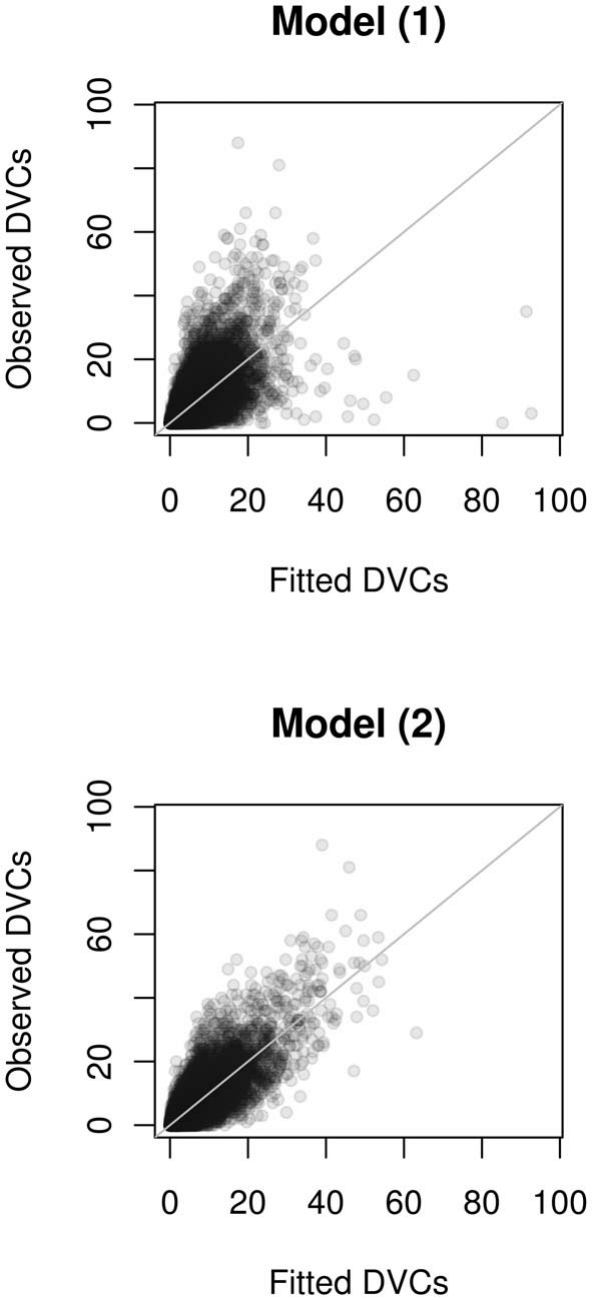
Goodness of fit. Goodness of fit for the parametric model (1) and the nonparametric model (2) illustrated by a scatterplot of the observed number of DVCs (ordinate) plotted against the fitted number of DVCs (abscissa).

The model fit of the final model (2) indicates that the model is able to discriminate between municipalities differing in the risk of DVCs (Fig. 2). The out-of-bootstrap log-likelihood (see Information S1) shows that the parametric model (1) without considering contributions of the environment (corresponding to zero boosting iterations in the diagram) is less accurate than the model considering environmental effects. Thus, climate, land use, and browsing variables contributed significantly to the final model.

### Assessment of collision risk

**Figure 3:**
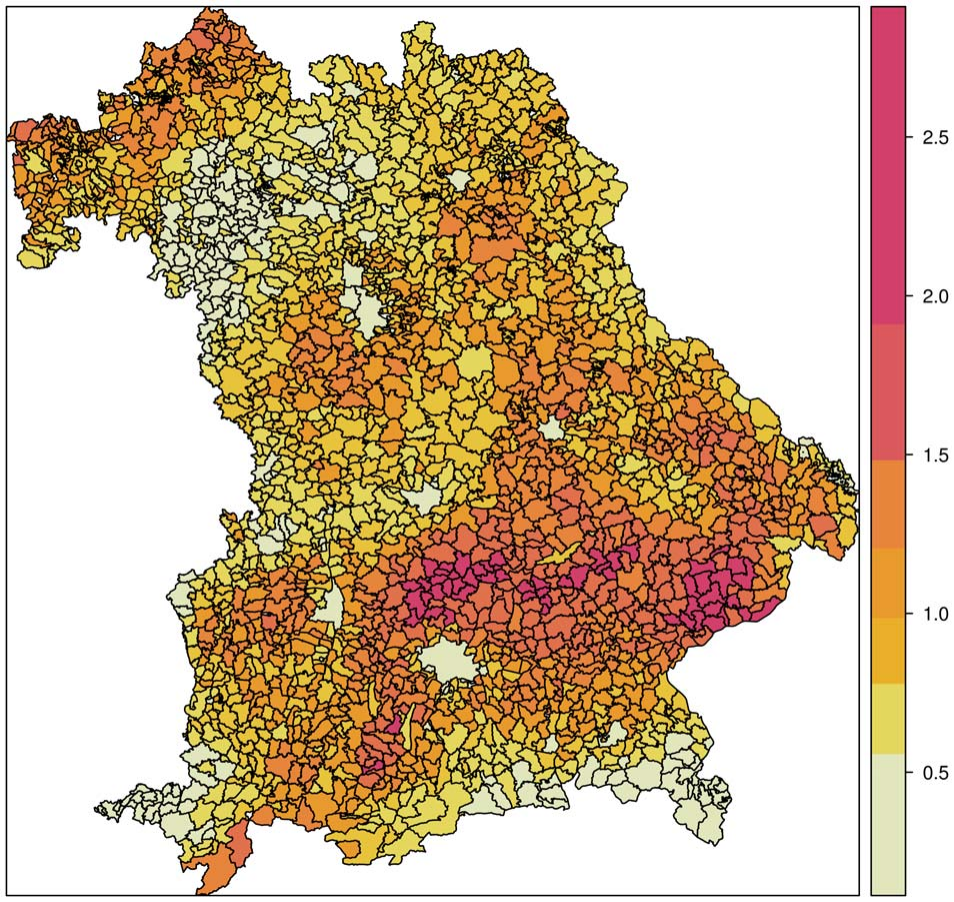
DVC index map. Collision risk, based on DVC index of climate, land use, browsing intensity, and spatial heterogeneity for 2006. The map shows seven risk classes based on the 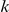-means classification method.

Taking all climate variables, land use variables, browsing intensities, and unexplained spatial heterogeneity simultaneously into account, we computed the DVC index for each of the Bavarian municipalities in 2006 (Fig. 3). For municipalities with an DVC index approximately equal to one, we can expect the same number of DVCs per kilometer as reported by the parametric model (1) in Fig. 1. A DVC index value of, e.g., 0.5, means that we only expect half of the number of DVCs per kilometer compared to the numbers reported in Fig. 1. In contrast, a larger DVC index value, e.g., 2.0, means that in this municipality twice as many DVCs per kilometer are to be expected. The map in Fig. 3 shows a general trend in the DVC index from unfavorable conditions for the roe deer in the Alps (southern border) and in the poorly productive, acidic mountains in the east to the lower elevations and productive soils south of the Danube River. We found a pronounced higher risk of DVCs south of the Danube River, where loess soils provide highly productive agriculture. Note that in some areas, the risk is more than twice as high as the baseline risk model (1). Larger cities such as Munich or Nuremberg were associated with small DVC index values.

Interestingly, the spatio-temporal model component 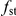 was not selected by stability selection for the final model (see Information S1). This means that the increased number of DVCs in 2009 compared to 2006 applies uniformly to all municipalities. In fact, the model suggested that the increase (most pronounced for secondary and tertiary roads, see Fig. 1) is due to an increase in the global risk of about 15%.

### Contributions of climate, land use, and browsing intensity

**Figure 4:**
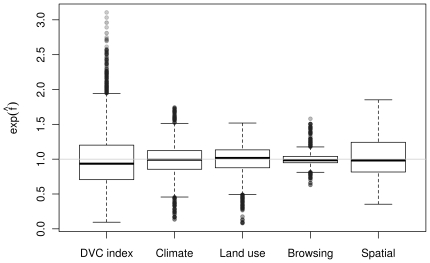
DVC index decomposition. Multiplicative decomposition of the DVC index into contributions of climate, land use, browsing intensity, and spatio-temporal terms.

The contributions of the different variables describing the environment can be decomposed owing to the multiplicative structure of the model on the exp scale. Figure 4 shows a decomposition of the DVC index. The plots revealed that climate contributed the most, followed by land use and space. Browsing intensity showed the lowest but still considerable influence (up to 50% risk increase; for spatial distributions of these ‘partial indices’, see Information S1).

**Figure 5:**
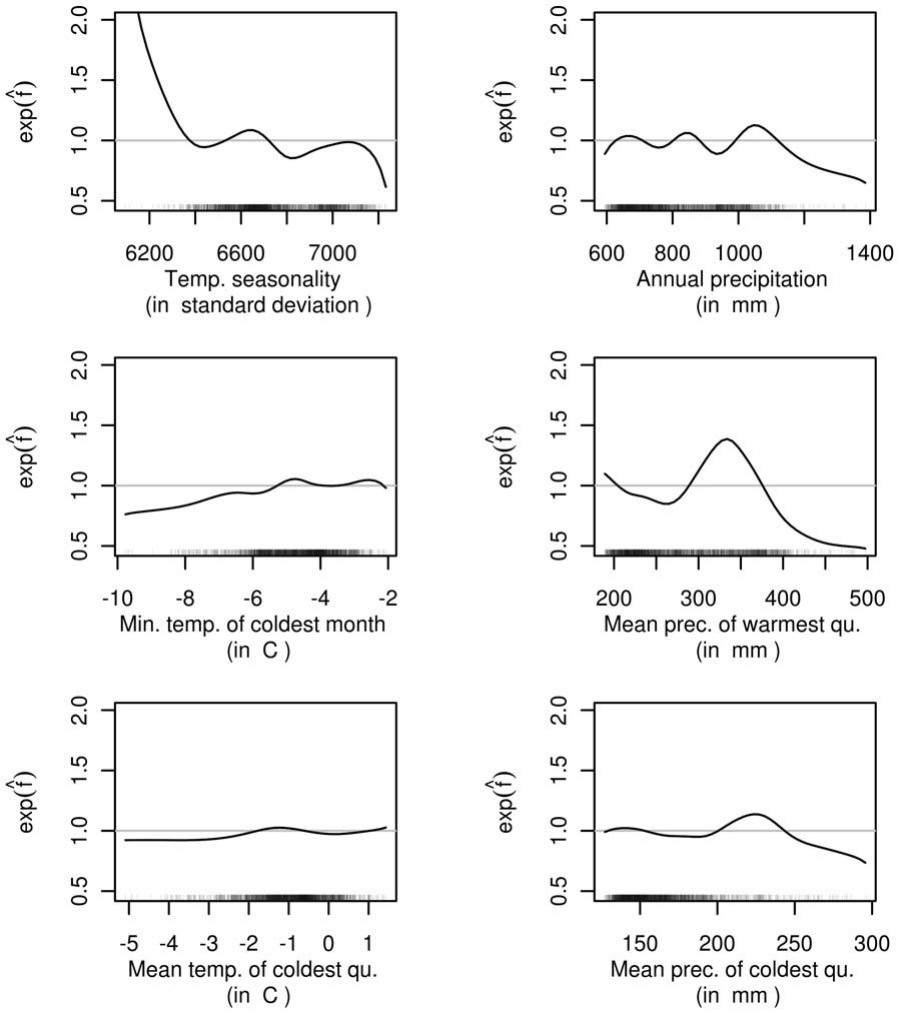
Effects of climate variables. Selected partial contributions of climate variables. The ordinate values can be interpreted as multiplicative changes in the expected number of DVCs per kilometer when all other variables remain constant.

We further decomposed the DVC index model into contributions of single environmental variables. Eight climate variables were selected by the stability selection procedure (see Information S1) and have an impact on the DVC index (Fig. 5). The mean and minimum temperatures in the coldest quarter or month had a positive impact on the DVC index (and thus on the expected number of DVCs per kilometer). The temperature seasonality appeared to have a negative influence, but the number of observations with values less than 6,400 was small. Precipitation in the warmest (summer precipitation) and coldest quarter (winter precipitation) showed clearly nonlinear effects, with a clear positive contribution for medium values. The effect of total annual precipitation was difficult to interpret owing to the erratic estimated function.

**Figure 6:**
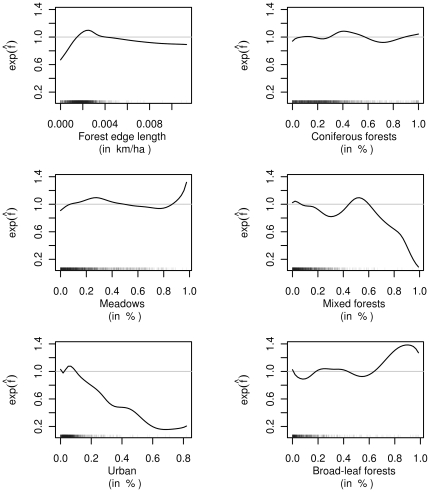
Effects of land use variables. Selected partial contributions of land use variables. The ordinate values can be interpreted as multiplicative changes in the expected number of DVCs per kilometer when all other variables remain constant.

Six land use variables were found to be influential (Fig. 6). We detected a clear nonlinear effect of forest-edge length, showing a steep increase up to medium values and a slight decrease for larger values. An increase in the proportion of urban area showed an almost linear decrease in the expected number of DVCs per kilometer. The proportion of meadows had a nonlinear impact on the expected number of DVCs per kilometer. An increasing amount of mixed forests decreased the accident risk, whereas a higher amount of coniferous forests increased the accident risk.

**Figure 7:**
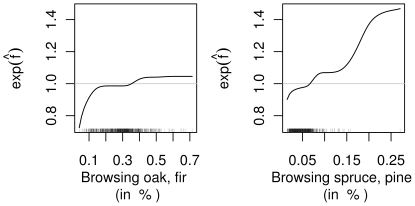
Effects of browsing variables. Selected partial contribution of browsing variables. The ordinate values can be interpreted as multiplicative changes in the expected number of DVCs per kilometer when all other variables remain constant.

Two of the four variables measuring browsing intensities were selected (Fig. 7). The expected number of DVCs per kilometer increased with the browsing intensity of the two highly palatable genera of trees, oak and fir. A reduction in risk of 20% was associated with municipalities in which only a small amount of saplings (less than 10%) showed signs of browsing on leading shoots. Spruce and pine are distributed in almost all forests in Bavaria but are less palatable for roe deer. However, a positive relationship between the proportion of browsed saplings and the expected number of DVCs can be inferred. The accident risk began to increase the baseline risk at a browsing proportion of approx. 7%. Note that the increased risk for municipalities with more than 15% browsing in spruce or pine is due to only a very few extreme observations.

### DVC index vs. harvest numbers

**Figure 8:**
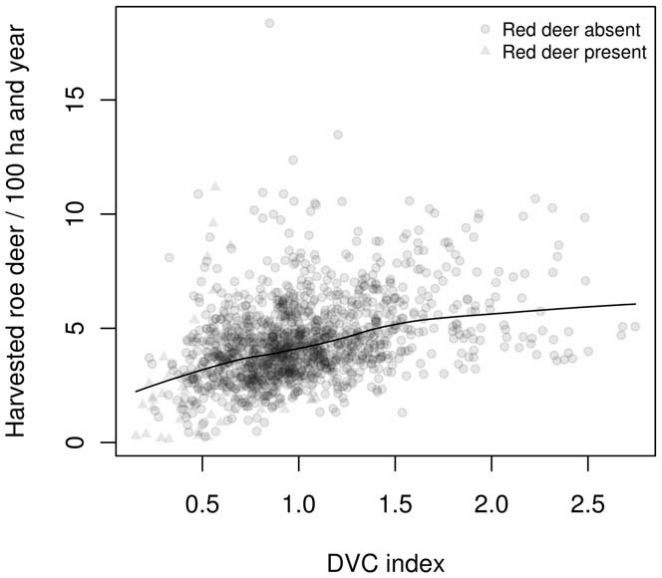
DVC index and harvest numbers. Comparison of DVC index (for 2006) and roe deer harvest numbers for 2004–2009 for each of the Bavarian game management districts. Mean regression was fitted by a LOWESS smoother.

To investigate whether the proposed DVC index can be interpreted as a relative measure for roe deer density, we compared the DVC index with roe deer harvest data reported for each of the game management districts. Figure 8 shows a scatterplot of the DVC index for 2006 and the number of roe deer harvested in the corresponding game management districts per 100 ha hunting area (excluding urban areas) and year (2004–2009 harvests shown simultaneously). Given the biases associated with harvest numbers and the lack of adjustment for hunting effort, the correlation between the DVC index and the harvest data was surprisingly large (Spearman rank correlation 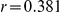, p-value 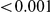). Interestingly, a more or less linear relationship was observed for DVC index values up to 1.5. For areas with more expected DVCs per kilometer (more than 50% increase), the harvest numbers remained more or less constant. In these game management districts, it should be possible to harvest up to ten roe deer per 100 ha per year. Very low roe deer harvest numbers were observed in red deer game management districts.

## Discussion

Using a novel modelling approach, we developed a high-resolution risk model for the number of roe-deer–vehicle collisions covering a large and densely populated area in Central Europe. From this model, we derived a spatially smooth map (Fig. 3) to assess the expected multiplicative change in the risk of DVCs within a specific municipality compared to the average state-wide risk for different road types. The issue of DVCs is of high relevance particularly in Central Europe and North America, and state agencies invest considerable financial resources for fencing and green bridges to decrease collisions with wildlife. However, risk models for collisions on a landscape scale have been developed only for the white-tailed deer and moose [21], [22], [19]. To our knowledge, only Seiler [11] has presented a risk model for collisions with roe deer, but only included four land use variables describing the environment. With our model, road management agencies can now differentiate between low, medium, and high DVC risk areas. Based on this easily communicable map, the limited resources available for road construction measures to avoid DVCs can now be assigned more efficiently to specific areas or roads.

The multiplicative structure of the contributions of variables describing climate, land use, and browsing intensity furthermore helps to identify possible causes for variations in the local risk. We could establish a clear but nonlinear connection between browsing intensity and the expected number of DVCs per kilometer, which supports the nonlinear relationship between fir regeneration and deer densities observed by Tremblay et al. [16]. Browsing is the only variable in our model that can be directly manipulated [through altered hunting quota, 35]. Thus, in addition to fencing and green bridges, an effective management of roe deer is an option to reduce DVCs. However, simply increasing harvest numbers is not appropriate. Sex-specific deer management may also be crucial, as indicated by an analysis of vehicle collisions with white-tailed deer in Ohio by Schwabe et al. [46], which demonstrated a reduced DVC risk in areas with a high harvest of does but an increased risk in areas with a high harvest of bucks.

We want to stress that the increase of DVCs with increasing browsing suggests that DVCs may be used as a simple proxy for roe deer densities on the considered scale of municipalities. Some French départements [2] already use this information for management, and the same has been proposed for Sweden [11]. Roe deer management in Bavaria is currently based on an assessment of browsing intensities. But browsing intensity can only reliably be measured in forested areas (roughly 1/3 of Bavaria) and also depends on the local tree species composition. Our approach using the DVC index provides a more comprehensive tool that can be used across a diverse landscape. The moderate positive correlation of the DVC index with harvest data support this fact. Nevertheless, a more convincing external validation of the DVC index is necessary. The next Bavarian browsing survey will take place in 2012, and a comparison of the DVC index for 2012 with the number of DVCs in each of the Bavarian municipalities that will occur during that year will give more insights into the usefulness of our index.

Our model is not only of interest for risk assessment, but also for investigating the main environmental drivers of this risk. Previous studies have identified climate and land use variables as important habitat variables for roe and white-tailed deer [47], [48], [25] and so does our model (see Fig. 4). Precipitation has been reported to have a negative effect on animal performance, particularly in winter and spring, with an overall negative effect on density [26]. It has been shown several times that deer density increases with productivity [27], [25]. The negative influence of increasing seasonality on the DVC index is similar to the general trend of decreasing deer densities towards continental conditions with higher seasonality [25]. Finally, an increasing length of forest edges positively influenced the DVC index, which is in line with many studies that revealed that increasing the length of forest edges increases the habitat quality for browsers, with short distances between resting and feeding places [28], [19], [49].

Although the effects of climate and land use are in line with published results on ecological drivers for roe deer abundance, the observed discrepancies can possibly be explained by our modelling approach, which differs considerably from published studies. Instead of directly modelling the–possibly transformed–DVC density by a linear model, our approach is based on a Poisson assumption for the number of DVCs and allows for nonlinearities. We furthermore differentiate between different road types; almost all previously published studies assume that the risk on motorways and smaller roads is the same. We also explicitly modelled the spatial autocorrelation that is certainly present in DVC data owing to its spatial nature. Last but not least, lower human population density in Scandinavia and North America may be one explanation for the different patterns in the effect of urban population on the number of DVCs. In contrast to Iverson [50], Seiler [11], and Farrell and Tappe [19] but in agreement with Bashore et al. [28], we found that a larger proportion of urban areas decreases the habitat suitability, and thus that available habitats for deer decrease with increasing populations of humans.

Although the results on climate, land use, and browsing variables make sense and are in overall good agreement with established knowledge, a considerable amount of heterogeneity in DVCs cannot be attributed to specific variables. The spatial variability in our model is high (Fig. 4) and shows clear patterns (see Information S1). The sources of these effects cannot be explained with our model, and one can only speculate about their nature.

Our newly proposed roe-deer–vehicle collision index provides valuable information about roe deer populations and a foundation for obtaining more precise ecological information on the deer. For private hunters aiming at leasing a hunting district and for land owners, the DVC index provides a basis for a fair agreement on the economic value of the rent. Furthermore, the establishment of a continuous monitoring system based on already available and spatially precise reports on DVCs is a cost-effective and helpful management tool for road and landscape planning, assessment of human health and insurance risks, and implementation of deer management plans. In association with information on browsing intensities and possibly other measures of population performance, our modelling approach provides means towards substantial improvements in deer management. Furthermore, the model framework applied here is easily transferable to other wildlife–vehicle collisions, for example for monitoring the distribution of rare species or for studying the impact of roadkills on populations.

## Supporting Information

Information S1 **Details on data sources, data preprocessing, and the statistical analysis. Contains R code to independently reproduce the results reported in this paper.**
(PDF)
