# Supplementary material for: Large-Scale Model-Based Assessment of Deer-Vehicle Collision Risk
Source: PLoS One. 2012 Feb 16;7(2):e29510. doi: 10.1371/journal.pone.0029510 (PMC3281017; doi:10.1371/journal.pone.0029510)
Supplement: Information S1 — Details on data sources, data preprocessing, and the statistical analysis. Contains R code to independently reproduce the results reported in this paper. (PDF) [file pone.0029510.s001.pdf]

Supporting Information S1

Material and Methods

Study site, data sources, and data preprocessing

The study area is shown in Fig. S1 1. The municipalities (administrative areas in Bavaria) are depicted in Fig. S1 2. Browsing data and roe deer harvest numbers were available on the scale of game management districts (GMD, in German "Hegegemeinschaften") and are shown in Fig. S1 3.

Deer–vehicle collisions. Data on wildlife–vehicle collisions in Bavaria are maintained in a central database by the Bavarian State Home Office. The database contains standardized information on all accidents caused by wildlife crossings that have been reported to the local police. Note that not only records of actual collisions with wildlife are contained in the database but also records of accidents induced by wildlife, such as collision with a tree while trying to avoid a collision with deer. The data are grouped according to the following species or groups of species:

- hares and rabbits
- red, roe, and fallow deer
- wild boars
- foxes
- badgers
- birds of prey, game birds
- other animals

Our analysis is based on deer–vehicle collisions (DVCs) involving only red, roe, or fallow deer. The following information about each accident is available:

- date and time

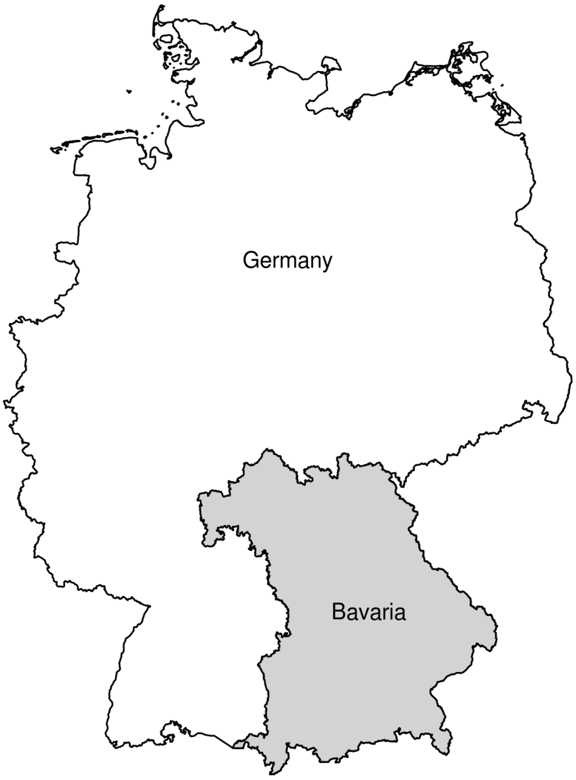

Figure S1 1. Map showing the federal state of Bavaria within Germany.

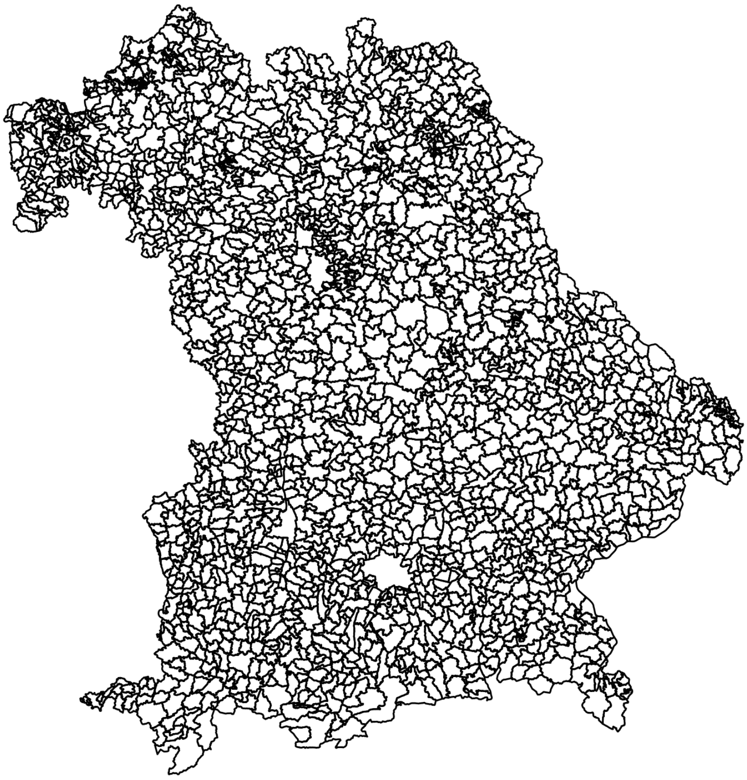

Figure S1 2. Borders of Bavarian municipalities.

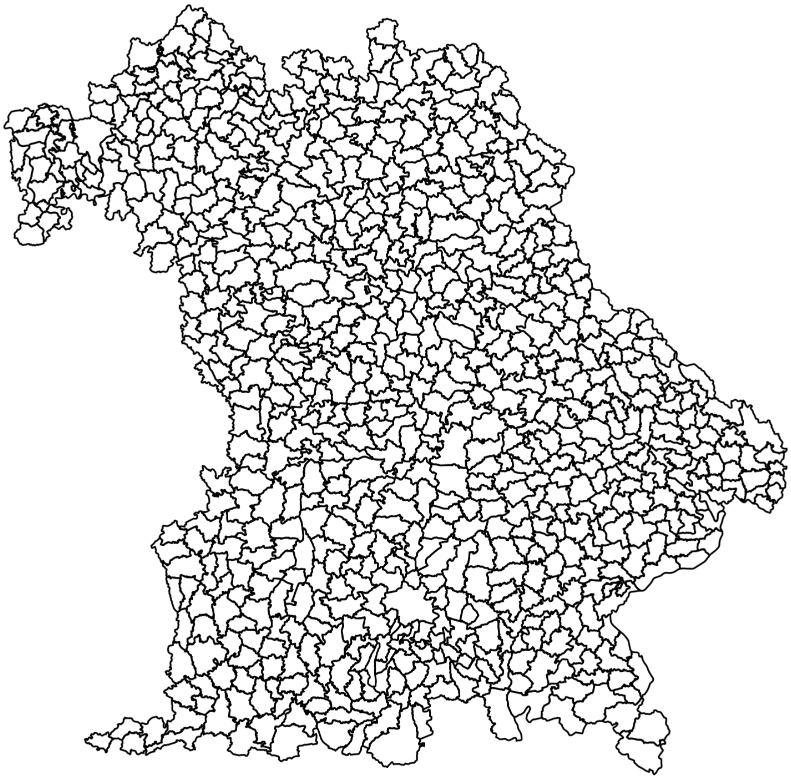

Figure S1 3. Borders of game management districts in Bavaria.

- identifier of municipality in which the accident was reported
- road type (motorway; primary, secondary, tertiary road; or residential street)
- road number (for motorways, and primary, secondary, and tertiary roads) or name of the residential street
- accident location as a chainage, i.e., distance in kilometers from a reference point, which is the point 0 for the particular road.

For all roads except residential streets, we computed Gauss-Krueger coordinates of the accident location based on the road number and chainage using the Bavarian Road Information System [1]. We first converted the chainages given in the database to the new station-based chainage system (i.e., a system with multiple reference points along one road, termed "OKSTRA"). In a second step, Gauss-Krueger coordinates were obtained [2].

Using points in polygon algorithms implemented in the GRASS geographical information system [3], the municipality in which each accident took place was determined. Most accidents occurred in the municipality in which they were reported. However, accidents that took place in larger unpopulated areas (in German "gemeindefreie Gebiete") were usually reported in one of the neighboring municipalities. For our analyses, we assigned accidents to the municipality in which they actually occurred. Our final data set consists of the number of accidents (those involving multiple vehicles were counted only once) for each municipality that is crossed by any road, grouped according to the road type (motorway primary, secondary, or tertiary roads; and residential streets) and year (2006 and 2009).

The usefulness of DVC data could be improved by better documentation of the accidents. The accident location can be more precisely recorded when the new chainage system (OKSTRA) is implemented in the DVC database. It would be additionally helpful if the species involved in an accident and ideally the sex of the animal could be recorded precisely. Continuous access to DVC databases will allow a constant data flow to be established and thus timely information about management success.

Overall, the number of accidents in 2009 was higher than in 2006. Figure S1 4 shows

[1] `http://www.baysis.bayern.de/`

[2] `http://www.baysis.bayern.de/OkWS/koordinierung.aspx`

[3] `http://grass.fbk.eu/`

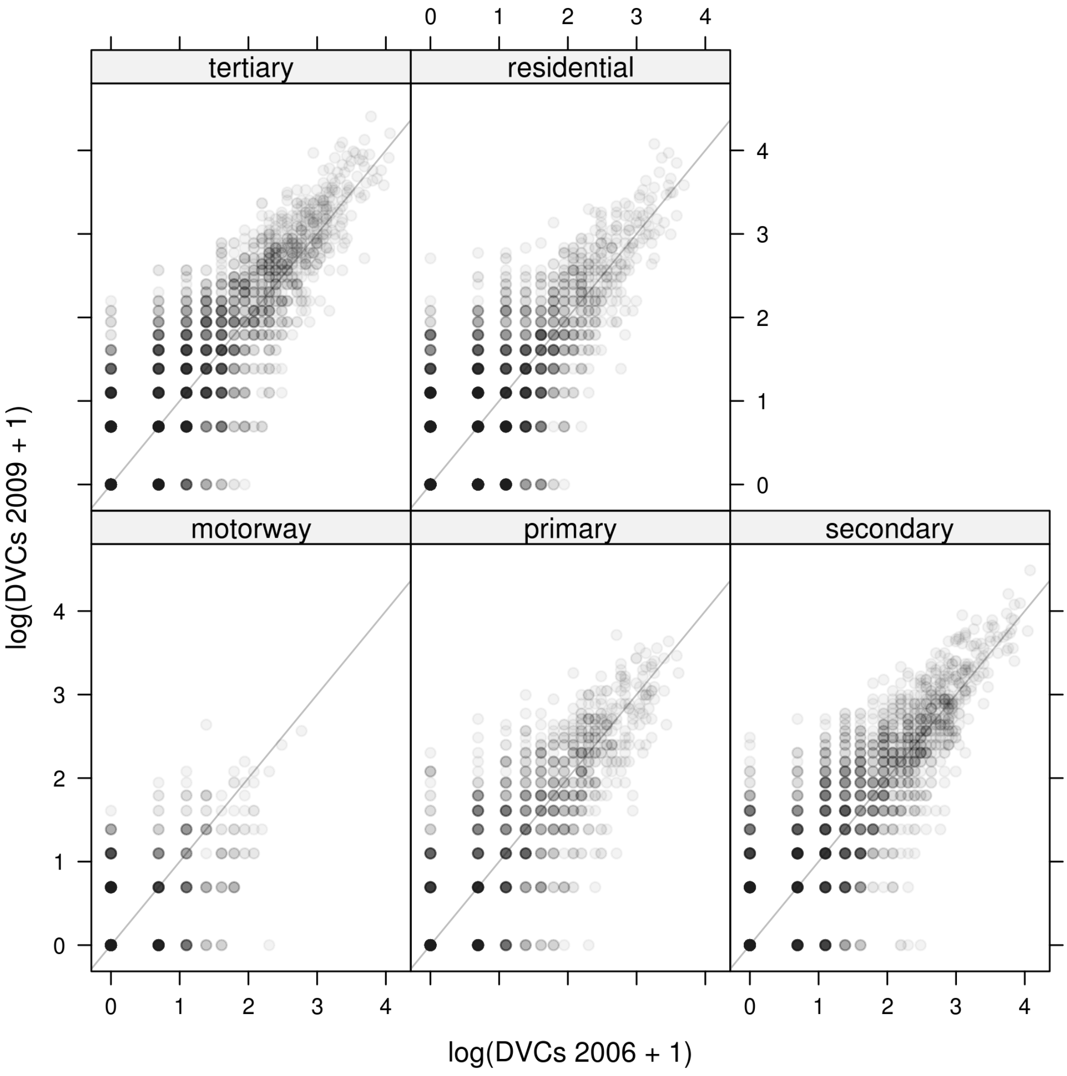

Figure S1 4. Number of deer–vehicle collisions (DVCs) in 2006 and 2009, grouped according to road type.

the number of accidents in 2006 and 2009 (as scatterplots) on the log scale, grouped according to road type. Except for motorways, there is a clear additive effect for all other road types.

The total lengths of the different road types within each municipality was computed from the official road map using GRASS. The results were checked against OpenStreetMap[4] data. As expected, the results were virtually equivalent for larger roads but not for residential streets. Our reported results are based on the official road map.

[4] `www.openstreetmap.org`

Climate and land use data. Based on the merged forest areas within a municipality, we calculated the total length of forest edges using FRAGSTAT 3.3 [5].

Combining classifications [5] from Corine, we derived the following relative coverages for each municipality: meadows (2.3.1., 2.4.3., 3.2.1), swamps (3.2.2., 4.1.1., 4.1.2), industry (1.2.1., 1.2.4., 1.3.1., 1.3.2.), urban areas (1.1.1., 1.1.2., 1.2.3., 1.3.3., 1.4.1, 1.4.2.), complex habitat (2.4.2., 2.2.2., 3.2.4., 2.2.1.), conifer forests (3.1.2.), mixed forests (3.1.3.), broadleaf forests (3.1.1.), and arable areas (2.1.1.).

Browsing data. The amount of ungulate browsing in Bavaria is monitored on a regular basis by the Bavarian Forest Administration [2, 3]. The number of trees in each grouping surveyed is given in Table S1 1. For each game management district and group of tree species, the proportion of browsed trees is reported by the authorities as a measure of browsing intensity. Here, we used slightly smoothed estimates that take into account the status of trees in the neighboring game management districts.

Table S1 1

Number of trees surveyed in 2006 and 2009.

Species	2006	2009	$\sum$
Spruce, Pine	783,806	717,429	1,501,235
Oak, Fir	119,761	132,267	252,028
Beech, other Hardwood	410,601	439,134	849,735
Ash, Maple, Elm, Linden	230,051	254,182	484,233
$\sum$	1,544,219	1,543,012	3,087,231

The smoothed estimates of the browsing intensities were derived from the following logistic random effects model [the so-called Besag-York-Mollié model; 1]

$$\mathbb{P}(\text{Browsing}|\text{GMD}_i) = \beta_i + \gamma_i$$

where β_i is independent of β_j. The correlation between γ_i and γ_j depends on the neighboring structure of the game management districts i and j and is defined by a Markov random field. For game management districts lacking observations of the corresponding tree species, we imputed the missing values as follows. We added one single artificial observation (one unbrowsed tree) to the data for a game management

[5] nomenclature at `www.eea.europa.eu/publications/COR0-landcover`

district lacking this information; the corresponding effect is then defined by the distribution of browsed trees in the neighboring deer management districts. The model was fitted using the `mboost()` function from the **mboost** add-on package [4] to the R system for statistical computing [see also 6, for a boosting algorithm for fitting the Besag-York-Mollié model]. The differences between the 'official' raw percentages and the smoothed estimates are displayed in Figs. S1 5 and 6. For game management districts with a sufficient number of trees surveyed, the numbers coincided well.

Roe deer harvest numbers. The number of roe deer harvested in each of the game management districts was obtained from the Bavarian State Ministry of Food, Agriculture, and Forestry. Roe deer are managed by tri-annual management plans, and the figures are based on the management periods 2004–2006 and 2007–2009. The numbers are based on the bagged game reported by local hunters to the local hunting administration; however, physical proof is not required. Since it is a regulatory offence to shoot fewer or more roe deer than defined in the management plans, the reported numbers may suffer from both under- and over-reporting. We therefore excluded game management districts that reported no bagged game in either period. Figure S1 7 displays a scatterplot of roe deer harvest numbers in the periods 2004–2006 and 2007–2009.

Statistical analysis

The trajectories of the out-of-bootstrap Poisson log-likelihood (divided by the number of out-of-bootstrap observations) as a function of the number of boosting iterations are given in Fig. S1 8.

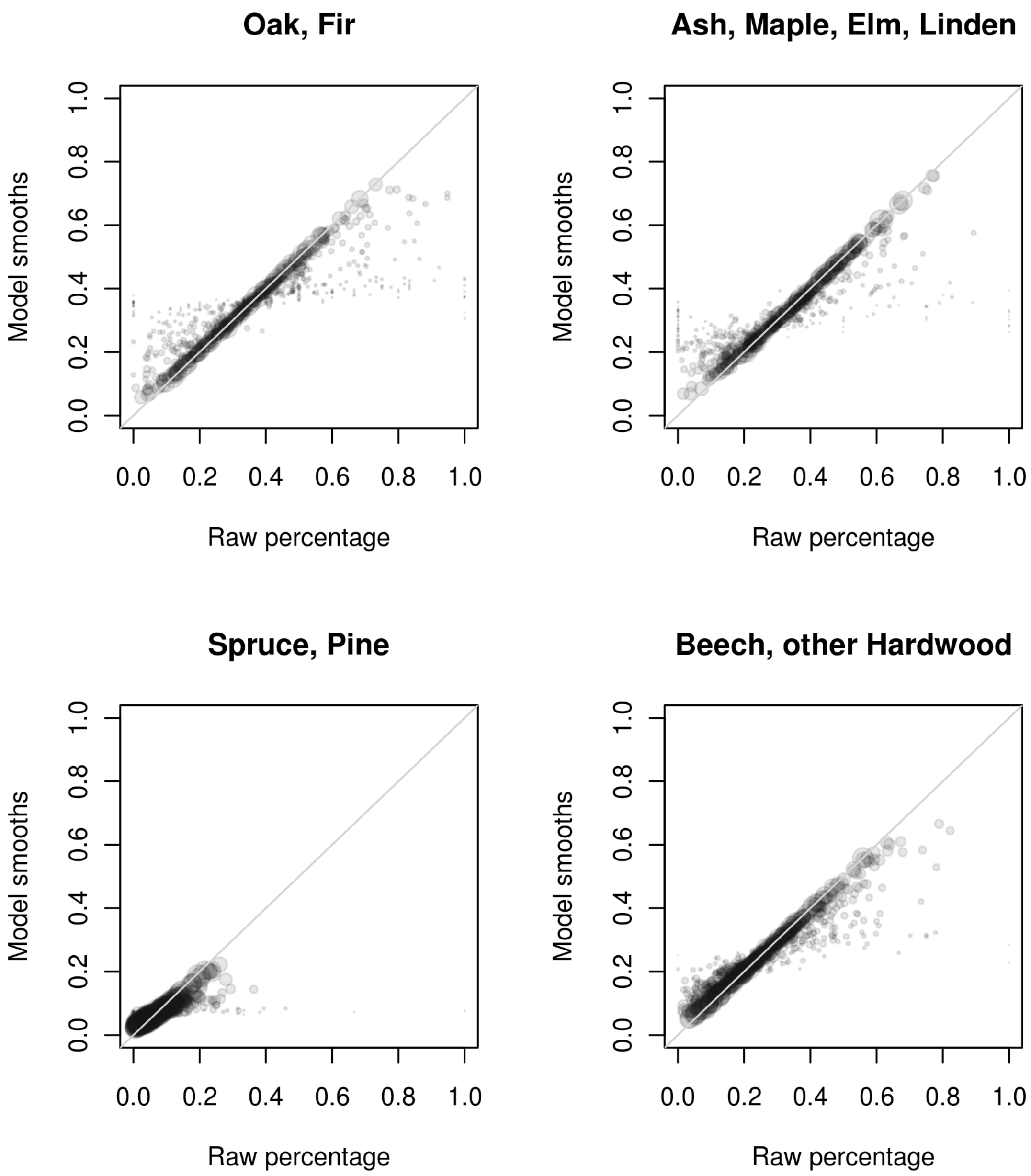

Figure S1 5. Differences between raw percentages as estimates of browsing intensities and smooth model-based estimates for 2006. The size of the dots is proportional to the square–root of the number of trees observed in the corresponding game management district.

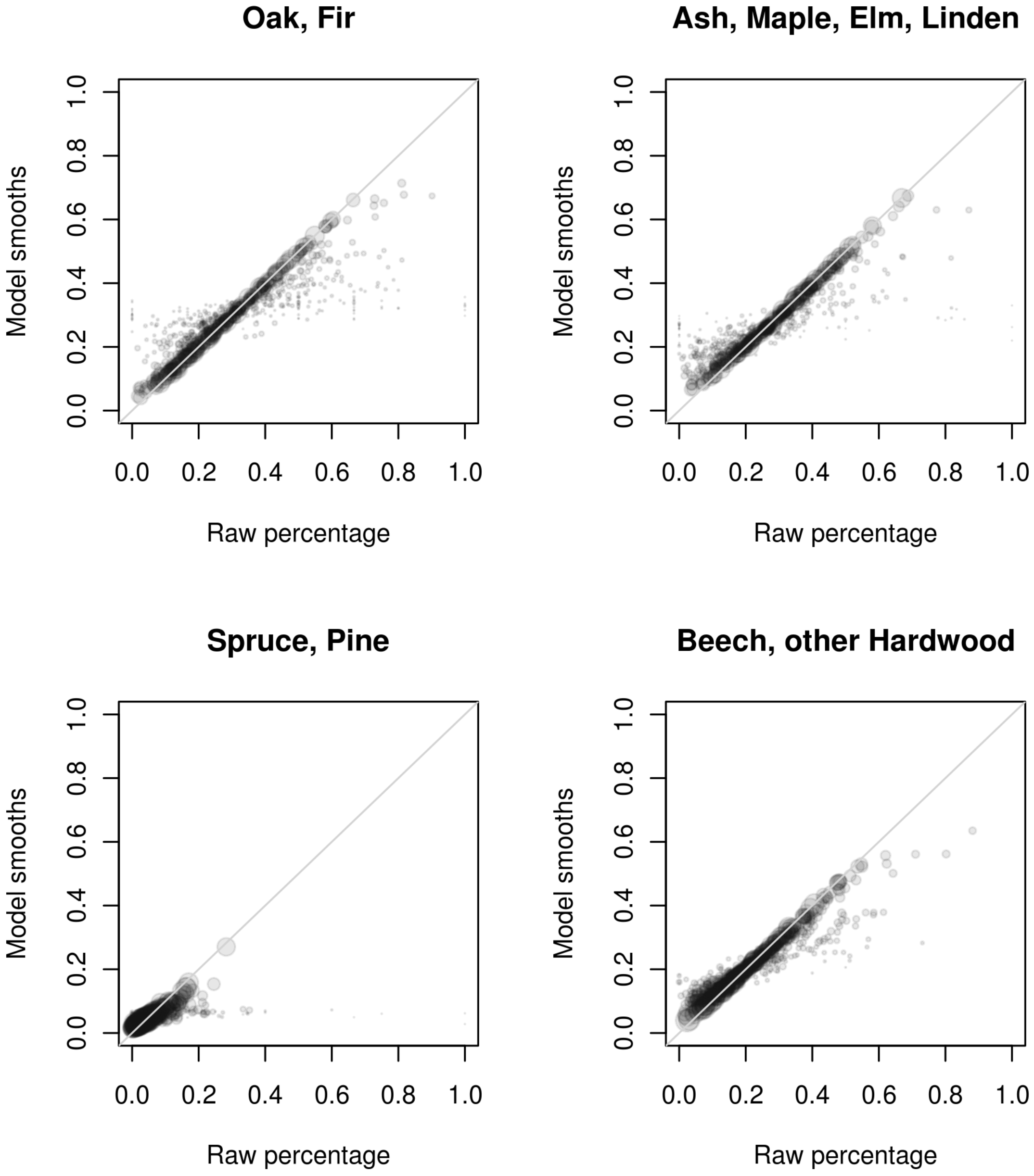

Figure S1 6. Differences between raw percentages as estimates of browsing intensities and smooth model-based estimates for 2009. The size of the dots is proportional to the square–root of the number of trees.

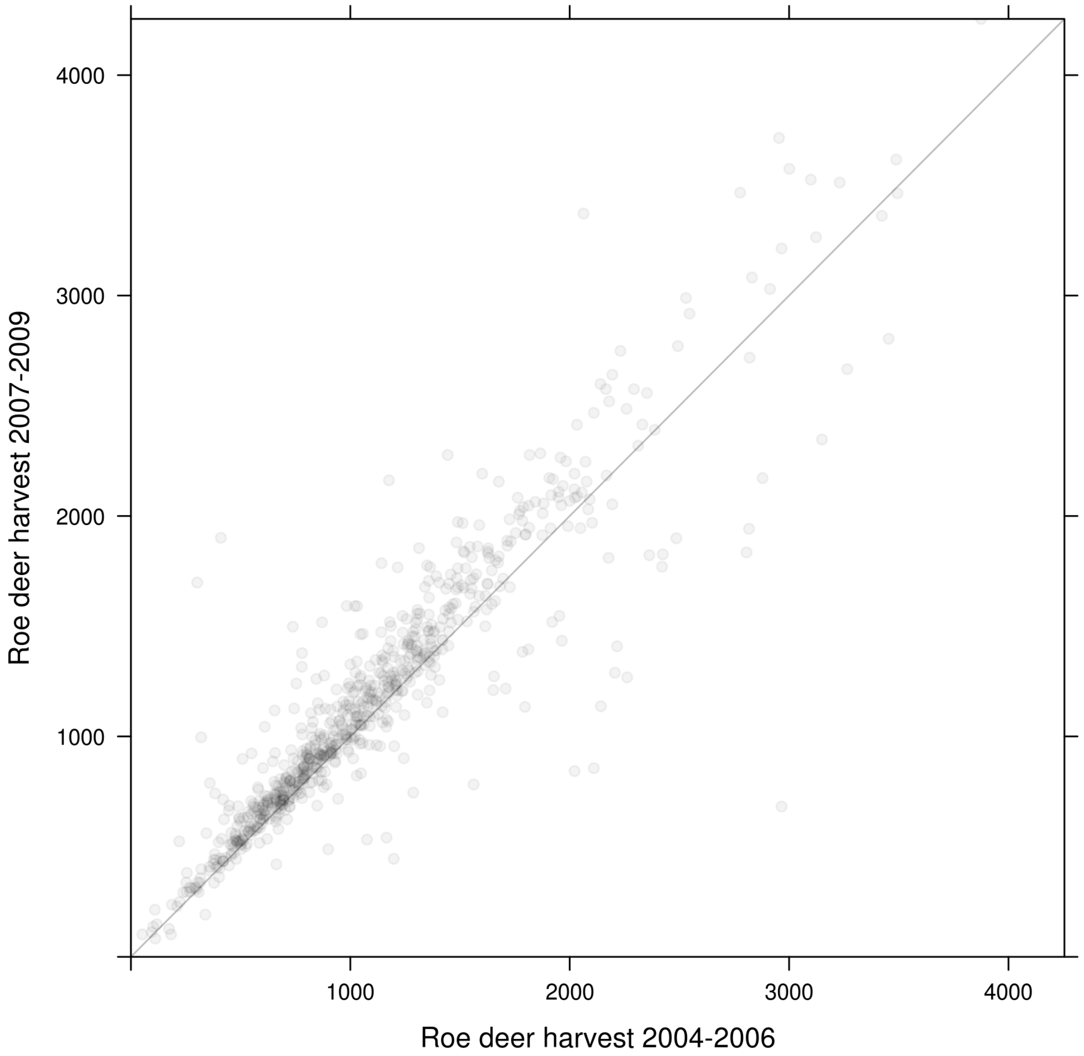

Figure S1 7. Number of roe deer harvests in the game management districts in the periods 2004–2006 and 2007–2009.

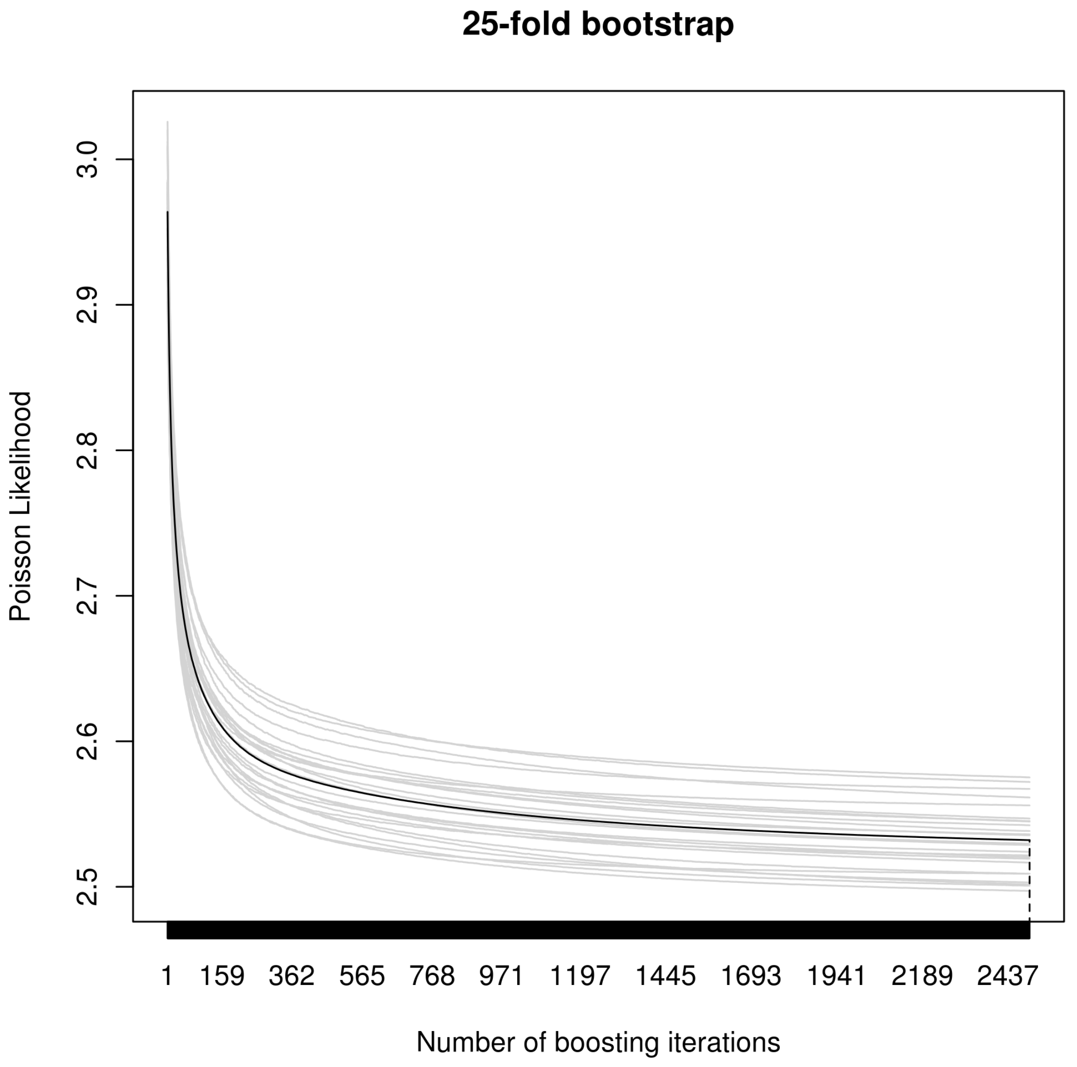

Figure S1 8. Out-of-bootstrap Poisson log-likelihood as a function of the number of boosting iterations (up to $2,500$).

Results

The following R code and data was used to fit models (1) and (2) by means of a Poisson linear model and model-based boosting [4]:

```
> ### attach boosting R add-on package
> if (!require("mboost"))
+     install.packages("mboost", repos = "http://R-forge.R-project.org")
> ### check for version number >= 2.1-0
> stopifnot(compareVersion(packageDescription("mboost")$Version,
+           "2.1-0") >= 0)
> ### load DVC data
> load(url(paste("http://www.stat.uni-muenchen.de/",
+                "~hothorn/data/BavarianDVCs.Rda", sep = "")))
> ### initialize RNG
> set.seed(29)
> ### climate
> bio <- colnames(rdata)[grep("^B_", colnames(rdata))]
> ### land use
> corine <- colnames(rdata)[grep("^C_", colnames(rdata))]
> ### browsing
> brow <- colnames(rdata)[grep("^b_", colnames(rdata))]
> ### set-up model formula
> fm <- paste("Freq ~ ",
+     ### unrestricted smooth functions
+     paste("bbs(", c(bio, corine), ", df = 6)", collapse = "+"), "+",
+     ### monotonic increasing
+     paste("bmono(", brow, ", df = 6)", collapse = "+"))
> ### spatio-temporal terms
> fm_XY <- as.formula(paste(fm,
+     "bspatial(POINT_X, POINT_Y, df = 6) +
+      bspatial(POINT_X, POINT_Y, df = 6, by = year)",
+      sep = "+"))
> ### fit baseline risk model (1)
```

```
> mod0 <- glm(Freq ~ offset(I(log(linelength))) + year + highway +
+             year:highway + Rotwild,
+             data = rdata, family = poisson())
> rdata$offset <- predict(mod0, type = "link")
> ### fit deviation from model (1), i.e., model (2)
> mod <- mboost(fm_XY, data = rdata, offset = rdata$offset,
+               family = Poisson(),
+               control = boost_control(nu = 0.025,
+                                       trace = TRUE,
+                                       mstop = 2500))
> ### cross-validation (mstop large, so no overfitting)
> cv <- cvrisk(mod)
> plot(cv)
> mstop(cv)
> mod[mstop(cv)]
> ms <- mstop(mod)
> ### for stability selection, mstop = 500
> mod[500]
> ### stratify subsampling weights by grid cell
> i <- factor(rdata$AGS)
> folds <- cv(rep(1, nlevels(i)), type = "subsampling")
> folds <- folds[i,]
> ### stability selection
> ss <- stabsel(mod, q = 20, folds = folds)
> ### reset mstop
> mod[ms]
> ### save results
> save(mod0, mod, ss, rdata, file = "model.Rda")
>
```

The selection frequencies obtained via the stability selection procedure are given in Table S1 2.

The contributions of climate, land use, browsing, and spatial heterogeneity to the DVC index index are shown in Figs. S1 9, S1 10, S1 11, and S1 12.

Table S1 2

Selection frequencies and ranking of the variables derived from stability selection criteria. We report only those variables with selection frequency greater than 0.9.

Variable	Selection Probability	Rank
Annual mean temp.	0.84	18
Temp. seasonality	0.96	11
Min. temp. of coldest month	1.00	1
Temp. annual range	0.88	16
Mean temp. of warmest qu.	0.08	24
Mean temp. of coldest qu.	0.92	14
Annual precipitation	1.00	1
Mean prec. of warmest qu.	1.00	1
Mean prec. of coldest qu.	0.96	11
Forest edge length	1.00	1
Meadows	0.96	11
Wetlands	0.24	23
Industry	0.88	16
Urban	1.00	1
Complex	0.76	19
Coniferous forests	0.92	14
Mixed forests	1.00	1
Broad-leaf forests	1.00	1
Arable	0.56	21
Browsing ash, maple, elm, linden	0.68	20
Browsing beech, other hardwood	0.00	25
Browsing oak, fir	1.00	1
Browsing spruce, pine	1.00	1
Spatial	1.00	1
Spatio-temporal	0.36	22

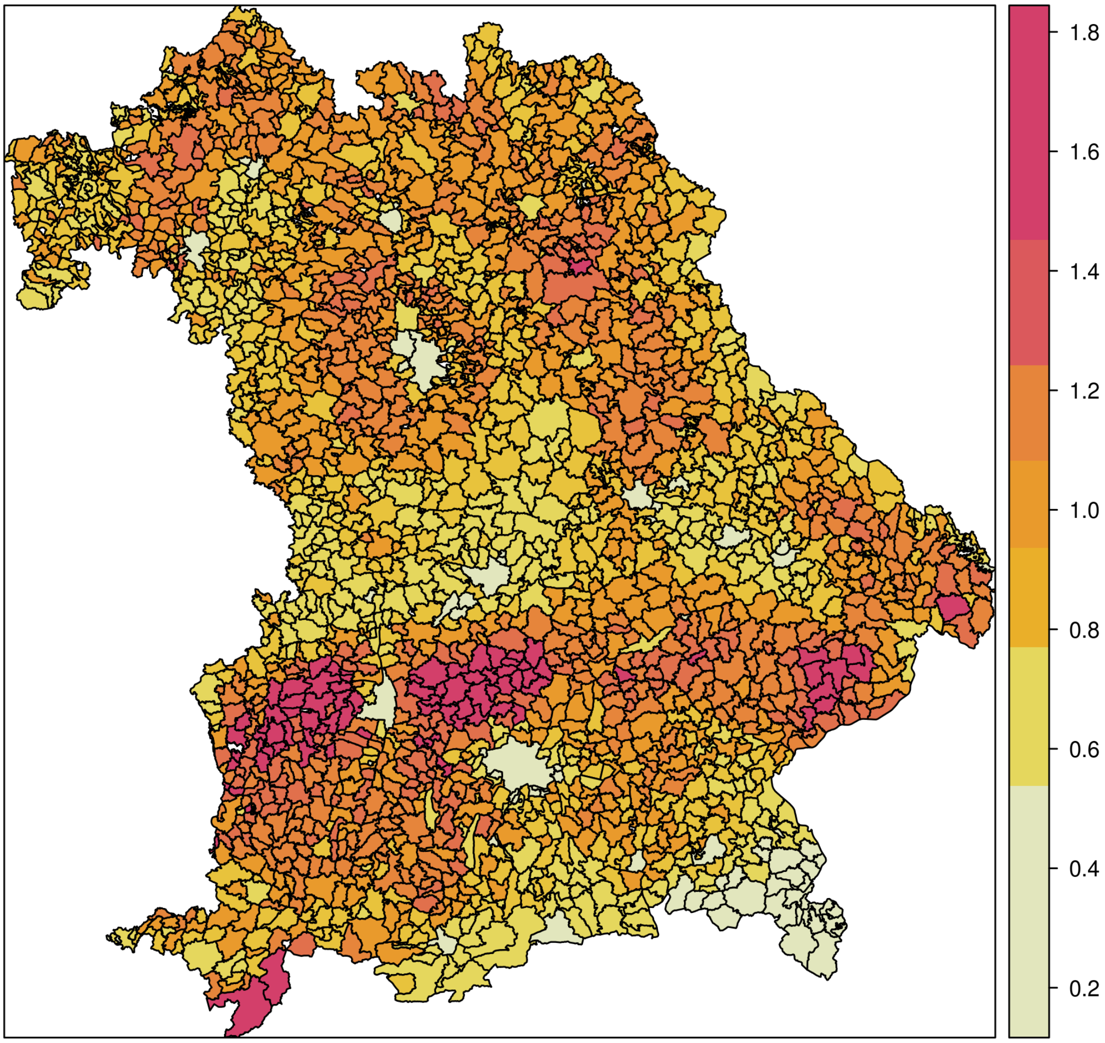

Figure S1 9. Contribution of climate and land use variables to the DVC index. The map shows the classification into seven risk classes based on the k-means classification method.

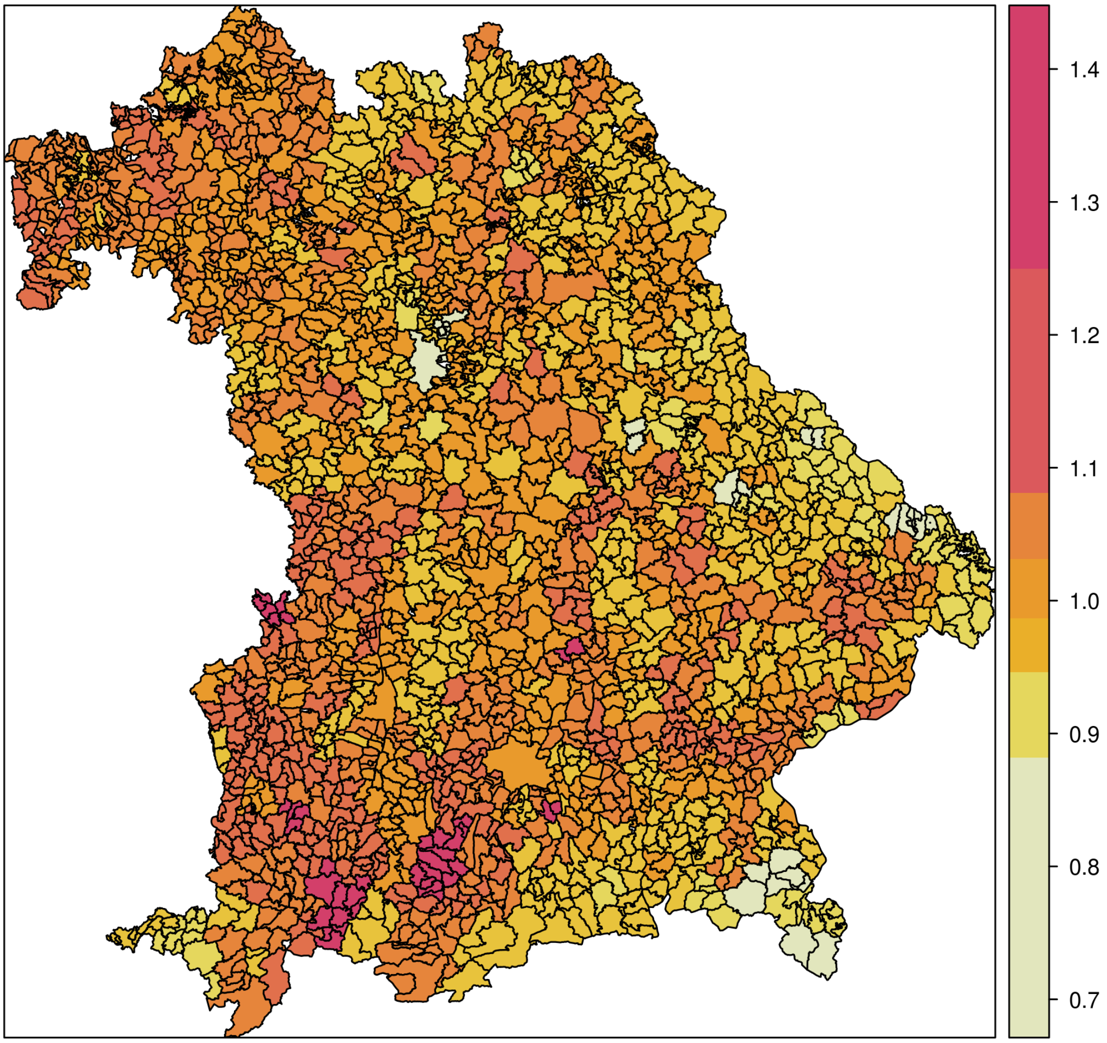

Figure S1 10. Contribution of browsing intensity to the DVC index. The map shows the classification into seven risk classes based on the *k*-means classification method.

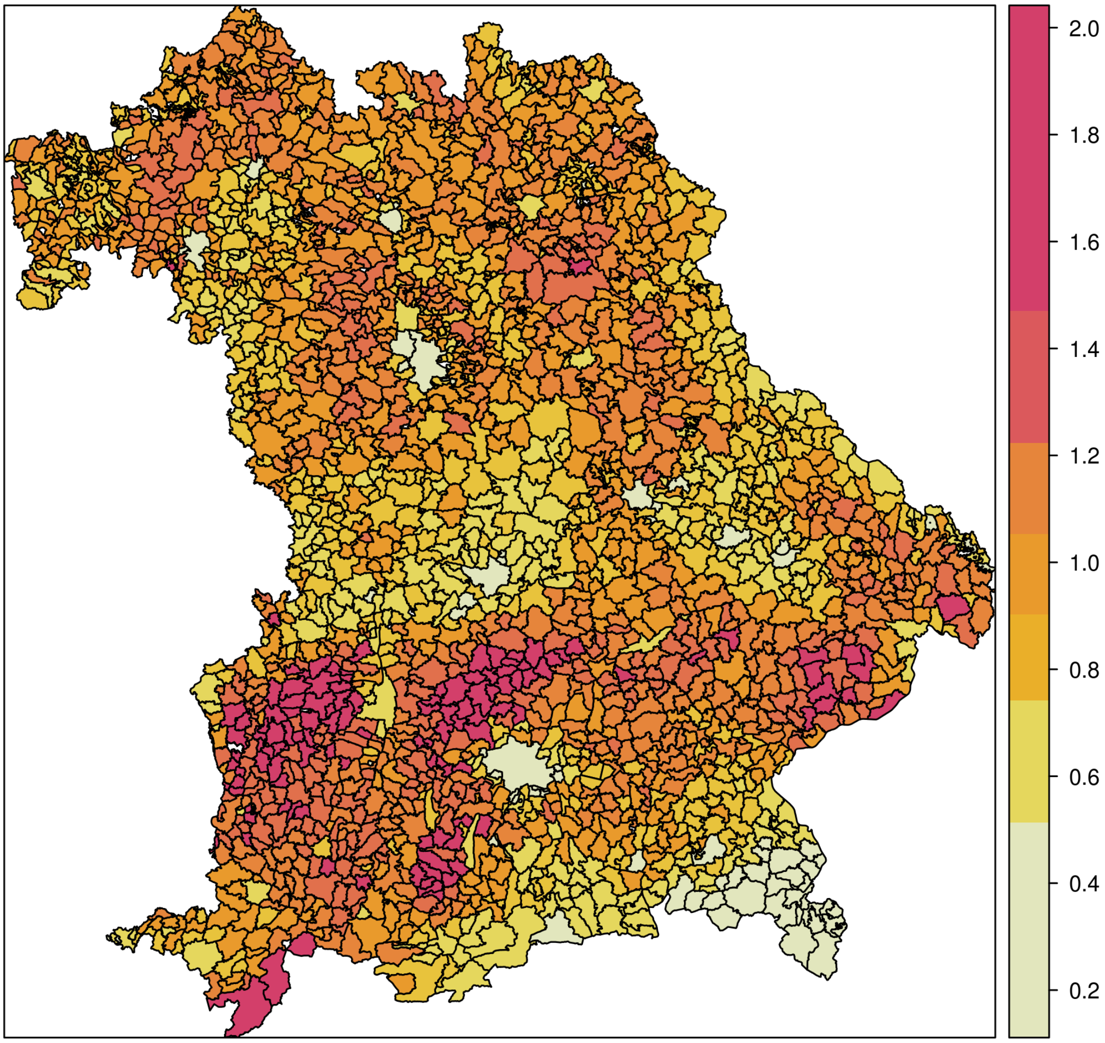

Figure S1 11. Contribution of climate, land use, and browsing variables to the DVC index. The map shows the classification into seven risk classes based on the k-means classification method.

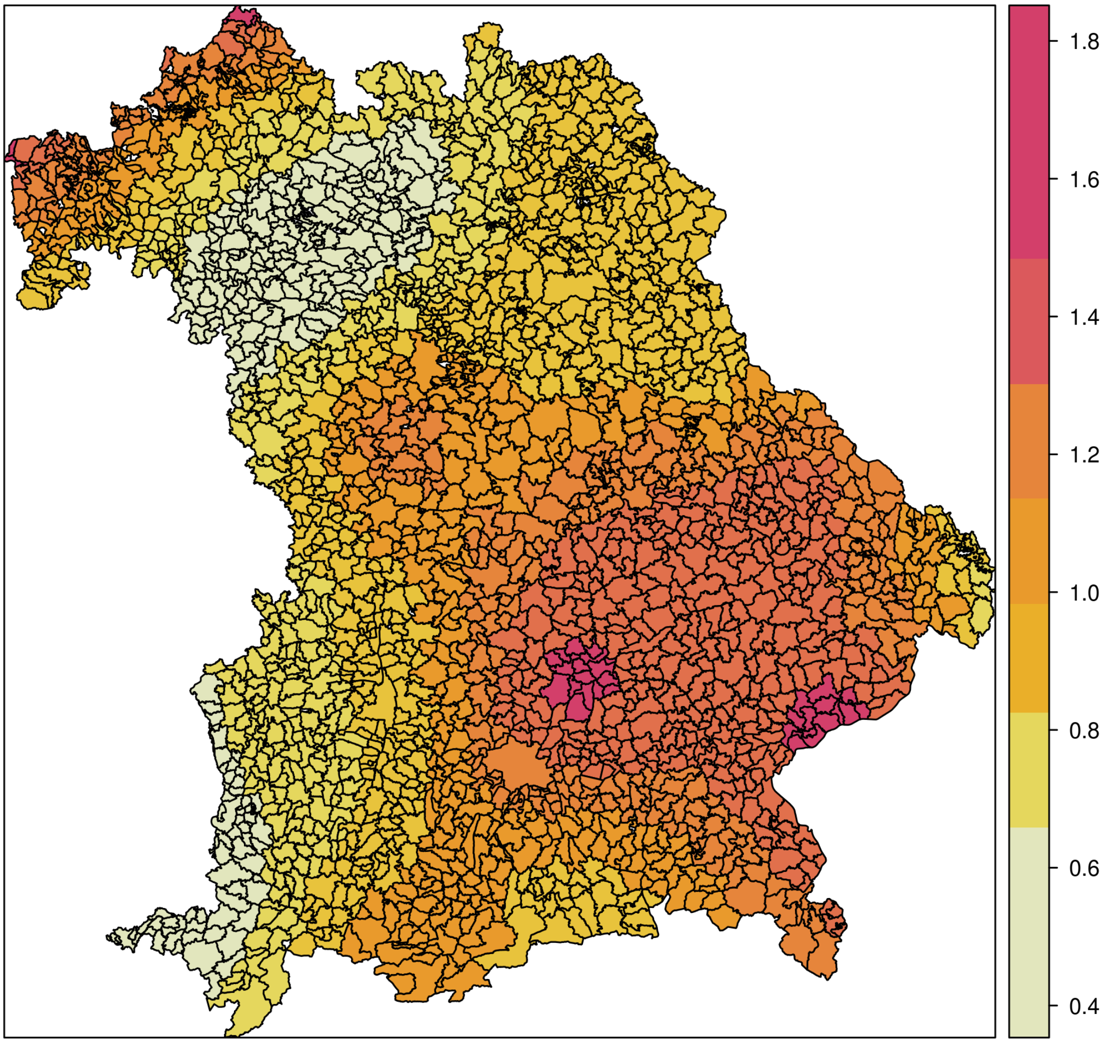

Figure S1 12. Spatial heterogeneity contributing to the DVC index. The map shows the classification into seven risk classes based on the k-means classification method.

References

[1] Besag J, York J, Mollié A (1991) Bayesian Image Restoration, With Two Applications in Spatial Statistics. Annals of the Institute of Statistical Mathematics 43: 1–59.

[2] Forstliches Gutachten (2006) Forstliche Gutachten zur Situation der Waldverjüngung 2006. Bayerisches Staatsministerium für Landwirtschaft und Forsten, URL `http://www.forst.bayern.de/jagd/verbissgutachten/`, accessed 2011-03-28.

[3] Forstliches Gutachten (2009) Forstliche Gutachten zur Situation der Waldverjüngung 2009. Bayerisches Staatsministerium für Landwirtschaft und Forsten, URL `http://www.forst.bayern.de/jagd/verbissgutachten/`, accessed 2011-03-28.

[4] Hothorn T, Bühlmann P, Kneib T, Schmid M, Hofner B (2011) Model-Based Boosting. URL `https://r-forge.r-project.org/projects/mboost/`, R package version 2.1-0.

[5] McGarigal K, Cushman S, Neel M, Ene E (2002) FRAGSTATS: Spatial Pattern Analysis Program for Categorical Maps. Computer software program produced by the authors at the University of Massachusetts, Amherst.

[6] Sobotka F, Kneib T (2011) Geoadditive Expectile Regression. Computational Statistics & Data Analysis Article in press.
